# Institutional Questions

**Published:** 1899-10-21

**Authors:** 


### INSTITUTIONAL QUESTIONS.

### Disinfection.

A correspondent writes: I shall be much obliged if you will explain what are the conditions under which sanitary authorities will undertake the disinfection of houses, &c., after an outbreak of scarlet fever or other infectious disease. I know of a family who have recently been put to much expense in consequence of one child developing scarlet fever in seaside lodgings, and there appears to be very general ignorance as to the extent to which the public authority may be called upon in matters of this kind.

You would do well to get an admirable little pamphlet, "Elements of Sanitary Law," by Alice Ravenhill, lecturer to the National Health Society, which sets out briefly and clearly many elementary facts in connection with sanitary laws which the general public would do well to master. (Published for the N.H.S. by the Sanitary Publishing Company, Limited, 5, Fetter Lane, E.C. Price 6d.) With regard to your question, the Public Health Acts provide that "every local authority may (in London must) provide disinfecting apparatus and may disinfect any articles free of charge, and compensate for unnecessary damage." Every local authority "can, on receiving a certificate from a legally qualified (*i.e.*, registered) medical practitioner, insist upon the owner or occupier of a house cleansing and disinfecting the same within a fixed time. . . . . If the order be not attended to the authority must do the work, and may recover the expenses in a summary manner from the owner or occupier in fault. Or in case of poverty, or other sufficient cause, the local authority may, with the consent of the owner or occupier, do the work at the public expense." The local authority in many cases will now "not only disinfect a house or room and its contents (free of charge when necessary), but make some allowance towards the expense of repapering; and some local authorities have wisely used their powers to provide temporary shelter for poor families compelled to leave their dwellings during the disinfecting process."

### The Plenum System of Ventilation.

"Architect" writes: Will you kindly tell me if the advantages and disadvantages of the system of ventilation known as the "Plenum" have been set forth and published in any form by its advocates and opponents, and if so where I may procure such publications?

The "Plenum" system is so far still on its trial, and though very strong opinions for and against its adoption for public buildings and hospitals are held by experts we do not think that much has been written on the subject. In a small pamphlet published by Messrs. Spottiswoode and Co., being a paper read before an architectural society by Mr. William Henman, A.R.I B.A,, the chief architectural exponent of the system in this country, you will find the matter briefly touched upon, and the contrary view is equally briefly mentioned in a paper by Mr. Keith D. Young, F.R.I.B.A., published by Messrs. Wyman and Sons, Great Queen Street, W.C. Sir Douglas Galton, in his book on "Hospital Construction," discusses the system at some length.

